# Unveiling a Hidden Threat: Wellens Syndrome as a Manifestation of Critical In-Stent Restenosis

**DOI:** 10.7759/cureus.93730

**Published:** 2025-10-02

**Authors:** John M Sousou, Ty J Merry, Christopher Marsalisi, Abhinav Karan, Pramod Reddy

**Affiliations:** 1 Internal Medicine, University of Florida College of Medicine – Jacksonville, Jacksonville, USA

**Keywords:** cardiology, interventional cardiologist, primary pci, wellens, wellens’s syndrome

## Abstract

Wellens syndrome is defined by characteristic T-wave changes seen on an electrocardiogram (ECG), indicating critical stenosis of the proximal or middle left anterior descending artery (LAD). Diagnosis is challenging, as patients often lack chest pain during ECG recording and have normal or mildly elevated troponin levels. Wellens syndrome classically arises de novo from a rupture of an atherosclerotic plaque, causing arterial obstruction. Although rare, some studies have reported Wellens syndrome secondary to in-stent restenosis (ISR). We present a case of Wellens syndrome caused by ISR of a previously implanted mid-LAD stent. The patient exhibited concerning ECG changes with new biphasic T-waves in leads V1 to V3, consistent with Wellens syndrome type A. Left heart catheterization revealed 80% ISR of his mid-LAD stent. We aim to highlight the critical importance of promptly recognizing the Wellens ECG pattern, not only in atherosclerotic disease but also in patients who have previously undergone stent placement.

## Introduction

Coronary artery disease (CAD) is the leading cause of death in the United States and is often referred to as a silent killer, as it can progress asymptomatically in many adults until the onset of acute coronary syndrome, a feared complication [1]. Wellens syndrome, also known as acute coronary T-wave syndrome, is the epitome of this reference as it may be “silent” to both patients and medical providers. Wellens syndrome is characterized by a distinct ECG pattern that typically occurs without other signs of ischemia, such as ST-segment elevations or pathological Q-waves, and often presents without the classic symptoms or ECG features of acute coronary syndrome.

Wellens syndrome can be classified as type A or type B based on ECG findings. Type A, observed in approximately 24% of cases, features biphasic T waves in the precordial leads V2 and V3. Type B, seen in about 76% of patients, is characterized by deeply inverted symmetric T-waves primarily seen in leads V2 and V3 [2]. Studies have shown that the appearance of the Wellens pattern had a high specificity (89%) and positive predictive value (86%) for severe stenosis of the left anterior descending artery (LAD) [3]. Wellens syndrome can present with ECG changes that overlap with those seen in other conditions, making accurate diagnosis and careful ECG interpretation crucial. For instance, coronary artery spasm, sometimes referred to as pseudo-Wellens syndrome, can mimic these ECG changes without the presence of significant atherosclerotic stenosis [2]. Additionally, myocardial bridging can produce similar ECG findings, especially during periods of increased myocardial oxygen demand [4]. 

As with other acute coronary syndromes, the timely recognition and therapeutic approach of Wellens syndrome is integral in the appropriate management of this condition, as it can indicate critical stenosis of the LAD. We describe the case of an asymptomatic patient with ECG changes consistent with Wellens syndrome who was found to have critical in-stent restenosis (ISR) of the mid-LAD.

## Case presentation

**Figure 1:**
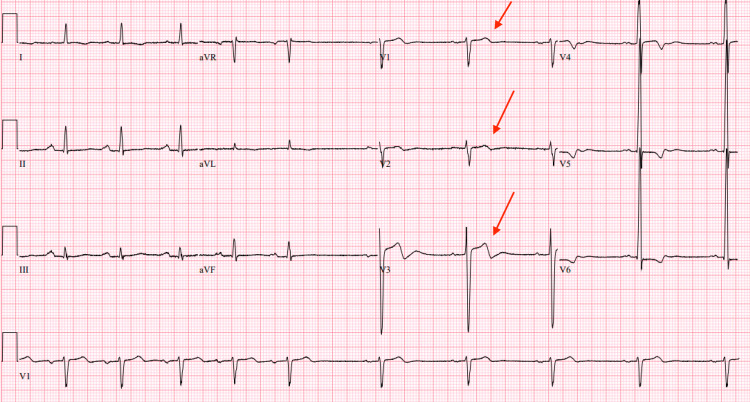
ECG showing biphasic T-waves with initial positivity and terminal negativity in leads V1, V2, and V3, consistent with Wellens syndrome type A (red arrows).

The patient is an 83-year-old man with a history of heart failure with reduced ejection fraction secondary to ischemic cardiomyopathy status-post percutaneous coronary intervention (PCI) to the mid-left anterior descending artery (LAD) four years prior, hypertension, and chronic obstructive pulmonary disease (COPD). The patient initially presented for worsening dyspnea with reported substernal chest pain on exertion and was admitted to the hospital for a COPD exacerbation. During his admission, an ECG was noted to have new biphasic T-waves in leads V1 to V3, consistent with Wellens syndrome type A (Figure 1). His troponin levels taken at hours zero, one, and three were all negative.

**Figure 2:**
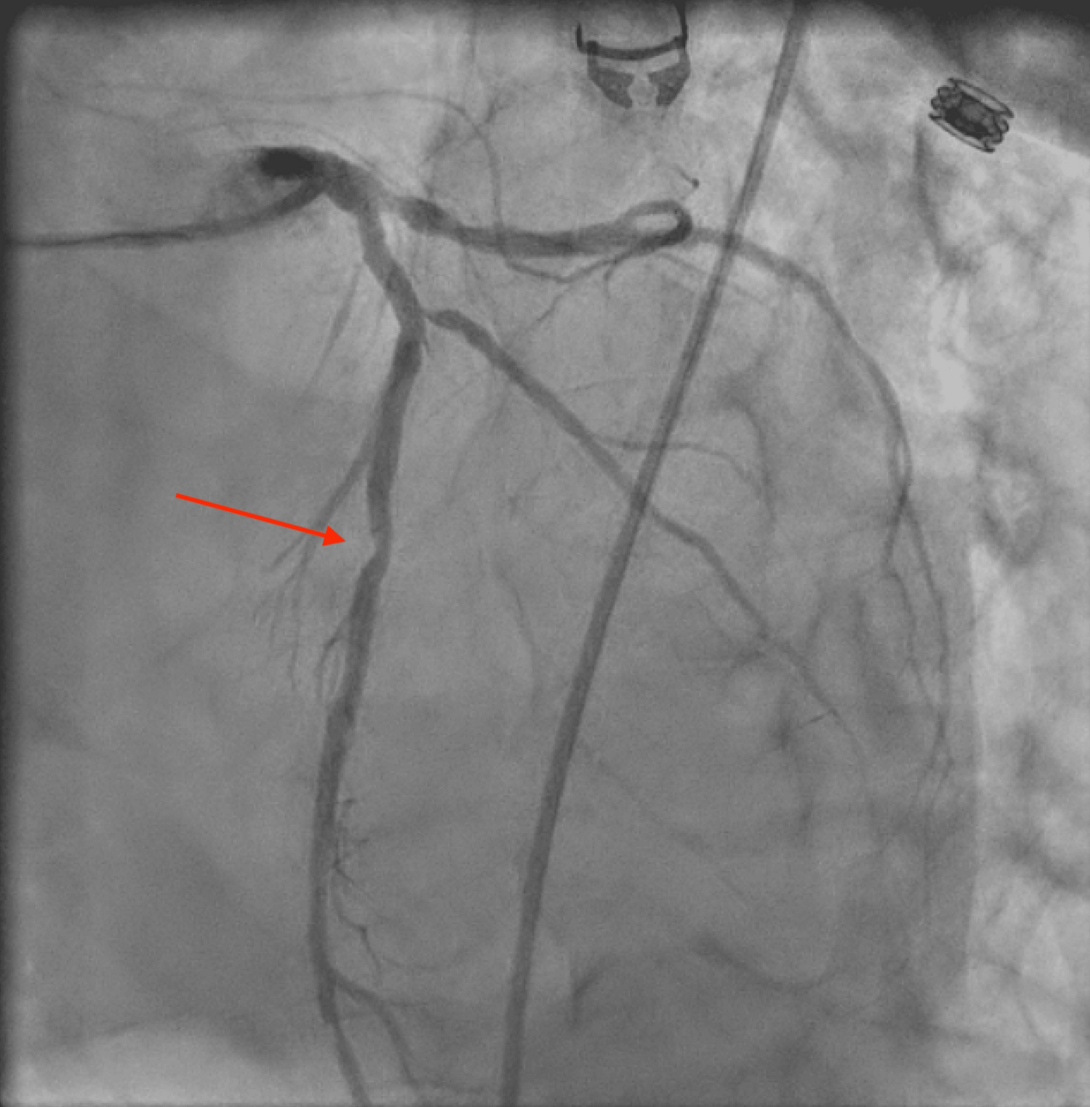
Coronary angiography revealing 80% ISR at the mid-segment of the LAD (red arrow).

The patient denied any chest pain throughout his hospital course but reported periods of angina prior to admission, specifically on exertion. He was ultimately taken for a left heart catheterization (LHC), which revealed 80% ISR of his previously placed stent in the mid-segment of the LAD (Figure 2). The patient underwent PCI with successful stenting of the mid-segment of the LAD at the bifurcation with the first diagonal artery (D1) using a 3.0 x 15mm Onyx Frontier drug-eluting stent (DES). The new stent was deployed in an overlapped fashion with the existing stent in the mid-LAD, and the existing stent was post-dilated using a balloon. Following PCI, the patient was initiated on triple therapy with aspirin, clopidogrel, and heparin, which was continued for three days until discharge. At the time of discharge, he was transitioned to dual antiplatelet therapy (DAPT) with aspirin and clopidogrel for a planned duration of one year. 

Our patient underwent initial follow-up with his primary care doctor and cardiologist within 14 days of hospital discharge, then routinely every six months. He remained adherent to DAPT through his one-year follow-up, at which point clopidogrel was discontinued. He has since continued to adhere to planned lifelong aspirin monotherapy. The patient has a higher chance of target lesion revascularization (TLS) after five years per the SORT OUT studies, which compared major adverse cardiac events and TLS [5]. Continuation of monitoring and routine EKG would be recommended in the outpatient setting to capture potential complications in the future.

## Discussion

Wellens syndrome represents a critical warning sign of severe proximal or mid-LAD stenosis, often signaling a pre-infarction state that requires prompt intervention. Like other forms of ACS, it shares common cardiovascular risk factors such as hypertension, diabetes mellitus, hyperlipidemia, tobacco smoking, and obesity [6]. Typically, the underlying pathophysiology involves rupture of an atherosclerotic plaque leading to abrupt arterial narrowing and impending myocardial infarction. In our case, however, the patient’s Wellens pattern was attributable to ISR, highlighting an important but less frequently discussed cause of LAD obstruction. Although Wellens syndrome is primarily linked to de novo stenosis of the LAD, there is evidence that it may, on rare occasions, arise from restenosis of a previously implanted stent [7]. There are several differences in the pathophysiology of Wellens between de novo coronary artery disease vs ISR. ISR is associated with biological and mechanical factors that promote stenosis or plaque rupture [8]. Exaggerated homeostatic healing in response to the arterial wall damage during stent implantation is a common biological response, whereas malposition or stent fracture is associated with mechanical pathology [8]. Based on the timeframe of our patient’s initial PCI, it could be theorized that the mechanism behind his ISR would be a malpositioned stent that may not have completely revascularized the site of the previous occlusion. ISR remains a clinically significant concern even years after PCI. Despite advancements in DES technology, restenosis rates persist, particularly among patients with complex lesions, diabetes mellitus, or a history of prior myocardial infarction. In our patient, the four-year interval between stent placement and ISR reflects the ongoing risk for restenosis even in patients seemingly stable on optimal medical therapy.

Our patient’s ECG displayed biphasic T-waves in leads V1 to V3, consistent with Wellens syndrome type A, where T-waves initially deflect positively before terminal negativity. Our patient met each of the criteria for Wellens syndrome, as shown in Table 1. 

Although Wellens syndrome type A primarily presents with biphasic T-waves in leads V2 and V3, studies have shown that biphasic T-waves may also be observed in leads V1 and V4 [9]. Similarly, the deep inverted T-waves seen in Wellens syndrome type B can occasionally extend from leads V1 to V4 [9]. Recognizing these patterns across a broader range of precordial leads can aid in the early detection and management of this high-risk condition. Additionally, Wellens syndrome is typically identified during asymptomatic periods or in patients with resolved chest pain. In our case, the patient denied active chest pain during ECG acquisition but reported occasional exertional angina prior to admission. This temporal dissociation between symptoms and ECG changes is another diagnostic hallmark noted in Wellens syndrome.

The patient’s ECG changes and clinical picture prompted us to pursue further ischemic workup with an LHC, given his cardiac history and prior PCI. The LHC revealed 80% stenosis of his previously placed stent in the mid-LAD, necessitating repeat PCI with stent placement. Management involves strict adherence to DAPT with aspirin and a P2Y12 inhibitor (such as clopidogrel) to reduce the risk of stent thrombosis and restenosis. Despite medical therapy, late ISR remains a threat, reinforcing the importance of timely follow-up care and continued risk factor modification for these patients.

**Table 1:** Diagnostic criteria for Wellens syndrome Credits: Created for the case report by John Sousou based on Nagdev et al. [10]. Permission was obtained from the original author.

Diagnostic Criteria for Wellens Syndrome
Type A: Biphasic T-waves in precordial leads (most commonly V2 and V3) OR Type B: Deep symmetric inverted T-waves in the precordial leads (typically V2 and V3)
Minimal or no ST segment elevation
Minimal of no elevation in cardiac enzymes
Prior history of angina but no chest pain during ECG
Sustained R-wave progression
Absence of pathological Q-waves

## Conclusions

Patients with new T-wave changes consistent with Wellens syndrome should undergo prompt ischemic evaluation, as this pattern indicates critical LAD stenosis. While literature on Wellens syndrome arising from de novo coronary lesions has been documented in various contexts, research on Wellens syndrome as a manifestation of ISR remains extremely sparse. Our goal is to elevate awareness and recognition among providers of the rare but possible presentation of Wellens syndrome in patients with a history of stent placement. 
